# Trajectories of health and social care expenditure in the last year of life among people 70 years and older in Region Stockholm: a population-based cohort study

**DOI:** 10.1186/s12877-025-06498-0

**Published:** 2025-11-04

**Authors:** Megan Doheny, Bo Burström, Janne Agerholm

**Affiliations:** 1 Aging Research Center, Department of Neurobiology, Care Sciences and Society, Karolinska Institutet, Stockholm, Sweden; 2 Department of Global Public Health, Karolinska Institutet, Stockholm, Sweden; 3 Centre for Epidemiology and Community Medicine, Stockholm County Council, Stockholm, Sweden

**Keywords:** Health and social care expenditure, Ageing population, End-of-life

## Abstract

**Background:**

Death is increasingly postponed to older ages which has implications for future care planning as health and social care expenditure peaks during the end-of-life. Hence, a detailed understanding of the care services involved is needed. This study aims to identify patterns of health-and-social care expenditure among people 70 years and older in their last year of life and to examine the associated socio-demographic factors.

**Methods:**

A population-based cohort study using linked register data, including those 70 years and older who died in Stockholm County in 2019 (*N* = 12,104). Health-and-social care expenditure was measured for the 365-days prior to date of death. A group-based trajectory model was used to identify common expenditure trajectories; the composition of care services and socio-demographic characteristics of the identified trajectories were assessed.

**Results:**

Six expenditure trajectories were identified that varied widely in the care services consumed before death. Most decedents followed a “persistently high” trajectory (36.5%) and had the highest average expenditure on health (in-and-outpatient) and home-help. The second largest trajectory (26.3%) was characterized as “early rise, persistently high”, who had the highest average expenditure on care home placements (SEK 532.0 K). Those in the lowest care expenditure trajectory were predominantly females in the lowest income quintile and born outside of Sweden.

**Conclusion:**

This study observed distinct trajectories of care expenditure among older people at the end-of-life and multiple factors contributed to these patterns. Most followed a trajectory where both where health and social expenditure remained high and persons relied on care in their own homes. These findings provide insight into the care resources used during the last year of life and may serve to inform future policies on care planning in the context of an ageing population.

**Supplementary Information:**

The online version contains supplementary material available at 10.1186/s12877-025-06498-0.

## Background

Contrary to popular belief, an ageing population does not automatically translate to a drastic increase in per capita healthcare expenditure, however, proximity to death does lead to an escalation of care expenditure [1, 2]. The utilisation of health and social care services peaks during the last year of life, and death is increasingly pushed to older ages [1]. In Sweden, over 80% of those who died in 2019 were 70 years and older, and the number of deaths occurring in this age group is increasing [3]. Generally, care expenditure among decedents is higher compared to survivors of a similar age [4]. Moreover, the increases in aggregate care expenditure observed during the last year of life are often not attributed to expensive life-saving treatments but rather to the high cost of caring for persons with multiple chronic conditions along with functional and cognitive limitations [5–8].

The older population at the end-of-life is increasingly diverse in terms of functional decline and morbidity, which is reflected in the wide variation in health and social care use and the subsequent care expenditure in the last year of life [6, 8–11]. Consistently, hospital-based inpatient care and social care services have been highlighted as the biggest drivers of high care expenditure at the end-of-life [7]. However, there is variation depending on age, as expenditure on inpatient care, specialist care and primary care decreases with older age, and expenditure on emergency care remains stable [4]. On the other hand, spending on social care services increases steeply with older age, as generally social care needs are concentrated in the last phase of life [4, 6]. Moreover, expenditure can vary depending care needs, institutional care (hereafter referred to as care homes) increases substantially among older adults who experience functional difficulties or have a dementia diagnosis [12], while spending on hospital-based inpatient care increases among persons with a cancer diagnosis [4]. Further, previous studies have observed that higher socio-economic groups incur higher healthcare expenditure over a lifetime, although lower socio-economic groups often experience poorer health comparatively [13, 14]. Such socio-economic differences in healthcare expenditure have been observed in the last year of life [15, 16]. A Swedish study observed that among decedents 65 years and older, those in the highest income quintile incurred higher inpatient care expenditure compared to those in the lowest income quintile. Additionally, inpatient care expenditure varied depending on social care use during the last year of life, where those living in their own homes with home-help incurred higher inpatient care expenditure than care home residents or those living independently without social care services [15].

In Sweden, the provision of health and social care is universal and primarily financed through taxes. Citizens only pay a small fraction out-of-pocket and there are cost ceilings in place which cap the maximum out-of-pocket costs. The provision of care is guided by the principle “*equal access for equal need*”, irrespective of an individual’s age, sex or economic resources [17, 18]. The organisation of care is divided between national, regional, and municipal levels. The national government mandates legislation and financial incentives, the 21 regions are responsible for financing and delivering health and medical care services, and the 290 municipalities are responsible for the financing and delivering of social care for older people including the provision of home-help services (personal care and domestic services) and the management of care homes [19]. Municipal social care is a single-entry system where older adults can apply for social care services from their municipality of residence and undergo a needs assessment to determine the type and level of services required. The provision of care for older people is guided by the principle of *“ageing in place*”, enabling older people to continue living in their own homes for as long as possible. Subsequently, the supply of municipal social care services has decreased in terms of the number of care home placements in recent decades [19, 20].

Increasingly, older people are relying on care and help in their own homes even in the last years of life. As the provision of health and social care services is fragmented in Sweden, it can be difficult for older people with multiple chronic conditions and complex care needs to manage care from several different providers. Poor coordination and communication between care services could increase the risk of older people having poorer experiences of care due to unnecessary care transitions, overtreatment and unmet care needs. These inefficiencies between the regions and the municipalities could lead to excessively high health and social care expenditure, during an already costly phase of life. Nevertheless, total formal care expenditure combining both health and social care has been less well examined in the Swedish context. Although previous studies examined the composition of care services involved at the end-of-life, the focus has been on either health or social care services [12, 15, 21, 22]. A greater understanding of the composition and interrelation between health and social care services involved in the caring of older people at the end-of-life, in addition to highlighting the care expenditure trajectories followed by older people can be valuable for future care planning. This study aims to identify trajectories of health and social care expenditure of people 70 years and older in their last year of life and to examine how socio-economic and demographic factors that are associated with these expenditure patterns.

## Methods

This is a population-based cohort study performed using individually linked register data (via encrypted serial numbers). The total population register “Registret över *totalbefolkning*” (RTB) was used to identify those 70 years and older registered as living in Stockholm County on the 31st of December 2018 which included (*N* = 269,122) persons [23]. This was linked to the Cause of Death Register to define the final study population (*N* = 12,104) as those who died during 2019. The year was selected to eliminate the impact of the COVID-19 pandemic. The registered date of death was used as the starting point to estimate monthly health and social care expenditure incurred by decedents during the 365 days prior to date of death.

Information to measure demographic factors was obtained from RTB. Sex was categorised into male or female, age was measured using year of birth, and country of birth was dichotomised as born in Sweden or other. The Longitudinal Integrated Database for Social Insurance and Labour Market Studies (LISA) is a collection of variables from several different population-based registers which are individually linked, where socio-economic and other measures were obtained [24]. Living situation was measured in the LISA register in 2018 and was based on a variable that categorises household composition, for the purposes of this study living situation was dichotomised to living alone or cohabiting (i.e. with a spouse, partner, another adult or a child under or older than 18 years) [24].

Income was measured using net annual equalised household income in 2017 from LISA and was ranked into quintiles from lowest to highest, based on the distribution of income among all inhabitants 18 years and older in Stockholm County. The approach of defining income quintiles based on the entire adult population was to situate older adults within the broader economic context in which they live and access services. Education level was categorised based on years of education according to Swedish educational system parameters: primary (< 9 years), secondary (9–12 years) and post-secondary (> 12 years).

Measures of underlying cause of death and place of death were retrieved from the Swedish Cause of Death register [25]. For the purposes of this study underlying cause of death was categorized into broad groups that represent the leading causes and fastest growing causes of death among older adults, i.e., cancer-related, cardiovascular-related, respiratory-related, dementia-related, and other (e.g. infections, accidents, or unintentional deaths) [26]. Place of death was categorized as hospital, care home or assisted living, private residence and other [25].

### Outcome

The last year of life was divided into monthly intervals. Health and social care expenditure was measured per individual for each interval, as the total and by the type of care including outpatient care (primary care, rehabilitation, occupational therapy), outpatient specialist care, emergency care, home-healthcare, inpatient care, home-help services and care home placements.

Healthcare expenditure was measured in the Region Stockholm healthcare utilization administrative database (VAL, by Swedish acronym) which is composed of several registers that contain information on all health and medical care financed by region Stockholm [27]. Healthcare expenditure was measured using reimbursement information retrieved in VAL between 2018 and 2019. All planned and unplanned inpatient care contacts (somatic, surgical, geriatric and psychiatric) were included to estimate inpatient care expenditure based on diagnostic related groups (DRGs). DRGs are assigned to each hospital stay based on primary diagnosis, patients age, procedures performed, and discharge status. DRGs reflect typical resource use profiles and are linked to standardized cost weights provided by the National Board of Health and Welfare yearly. DRG weights were multiplied by the monetary value per DRG unit adjusting for price level in 2019.

Measures of expenditure on outpatient care, emergency care, specialist outpatient care, and home-healthcare services were based on the registered cost weights attached to each care contact in VAL. The cost weights correspond to a standardized compensation to the care provider, which was multiplied by the fixed monetary value in 2019 [25]. Expenditure on privately provided specialist outpatient care was estimated based on the unit cost reimbursed by the region. Home-healthcare expenditure was calculated according to the type (basic or advanced) and length of provision. Cost weights were missing for 4% of all outpatient care contacts, and in these cases, expenditure was estimated by calculating the mode per contact based on the type of care, form of contact and whether the care activity was with a doctor or another care professional.

Social care expenditure was estimated using data retrieved from the Swedish Social Service Register (SOL) which collects data on publicly funded municipal social care services including home-help (domestic and/or personal care) and care home placements granted to individuals monthly [28]. Expenditure on home-help services was estimated by multiplying the number of hours of home-help granted to an individual per month, as measured in SOL, by the price per hour of home-help to the municipality. The average monthly cost per care home placement/other residential facilities was estimated based on data collected by “*Sveriges Kommuner och Regioner”.* Specifically, the national average net costs incurred by municipalities for providing care home placements/other assisted living accounting for income from internal transfers and external sales, divided by the number of permanent residents [29]. In 2019, the average municipal expenditure was 900,000 SEK per resident, and to obtain the per month cost this estimate was divided by twelve. This monthly estimate was combined with the number of months individuals spent in a care home prior to the date of death which was obtained from SOL [28].

## Analysis

A group-based trajectory modelling (GBTM) was performed to identify distinct sub-groups with similar trajectories of health and social care expenditure during the 12-months prior to the date of death [30–32]. Expenditure outcomes were transformed using the natural logarithmic scale to correct for distortions in the distribution and assigning zero to the transformed value if zero care expenditure was incurred by the individual in the respective month [33]. To determine the number of groups, models were constructed with (2–7 group) solutions with a combination of trajectory shapes (intercept only, linear, quadratic, cubic, quartic and quintic). The model selection process, the optimal number of groups and orders was determined by comparing the Bayesian Information Criterion, and classification of uncertainty indicated by Entropy. Moreover, models where < 5% of the study population were assigned to a group were excluded to ensure model stability and interpretability, even if fit indices were slightly better. The accuracy of the model was assessed using the average posterior probabilities assignment for each group (0.7 or higher), and the odds of correct classification (5.0 or higher), see Table S1. The mean expenditure on healthcare, social care and total care expenditure was assessed within 95% CI of the identified spending trajectories, see Table S2. The average per individual expenditure for health and social care services (expressed as SEK in thousands) and the demographic and socio-economic composition of the identified trajectories were examined descriptively.

Multinominal logistic regressions were performed to assess the factors associated with membership to care spending trajectories identified in the GBTM. This method was selected because the dependent variable spending trajectory is categorical with more than two unordered levels. The largest spending trajectory identified was set as the reference category (group 5, “persistently high”) and models estimated the probability of following another spending trajectory (compared to the reference) as unit change in the independent variables. Model 1 was adjusted for sex, age group, country of birth, living situation, and income group. Model 2 was adjusted similarly to model 1, with the addition of underlying causes of death and place of death. To assess the association between underlying cause of death, following an expenditure trajectory, the underlying causes of death were dichotomized, e.g., cancer-related vs. non-cancer-related) and places of death were similarly categorized (i.e. hospital vs. non-hospital deaths). This approach was selected to facilitate focused comparisons as well as aligning with the model assumptions of mutually exclusive categories, supporting model stability and interpretability. The estimates from models are presented as Relative Risk Ratio (RRR) within 95% confidence intervals (CIs). Stata version 15.1 (Stata Corp. College Station, TX) was used for statistical analyses.

## Results

The composition of study population (*N* = 12,104) is described in Table 1. The average age of decedents was 84.3 years, and the majority were women (53.9%). Most decedents were in the lowest income group (38.5%), had obtained secondary level education (41.6%), 79.9% were born in Sweden, and 69.8% were living alone. Almost a third of the decedents had a circulatory-related underlying cause of death and 23.1% were cancer-related, 17.4% were dementia-related and 7.2% were respiratory-related. Most deaths occurred in hospitals (44.3%), and 13.9% died in private residences.

**Table 1:** Description of the study population in socio-economic and demographic factors and the mean health and social care expenditure in the last year of life

	Total population	Mean expenditure in Swedish Kronor in thousands (SEK K)					
	*N* = 12,104	Healthcare		Social care		Total care	
	(%)	Mean(SEK K)	SE	Mean (SEK K)	SE	Mean (SEK K)	SE
*Age groups*							
70–74 yrs	13.7	371.1	(11.7)	132.5	(7.5)	503.5	(13.9)
75–79 yrs	16.5	293.5	(7.4)	150.6	(7.0)	444.0	(9.9)
80–84 yrs	19.1	249.2	(6.1)	241.2	(8.9)	490.4	(10.6)
85–89 yrs	21.8	202.8	(4.8)	317.0	(8.8)	519.8	(9.7)
≥90 yrs	28.9	142.5	(3.2)	453.5	(8.9)	595.9	(9.2)
*Sex*							
Male	46.1	264.6	(6.5)	223.7	(5.4)	488.2	(6.8)
Female	53.9	204.8	(3.4)	345.0	(6.0)	549.8	(6.5)
*Income groups*							
(lowest) group 1	38.5	199.9	(4.0)	332.6	(7.0)	532.4	(7.8)
group 2	35.0	237.8	(4.5)	271.0	(6.7)	508.8	(7.5)
group 3	11.4	267.4	(10.2)	251.5	(11.7)	518.9	(14.4)
group 4	6.4	283.3	(11.4)	239.7	(15.1)	523.0	(17.8)
(highest) group 5	8.5	273.8	(12.0)	256.7	(13.5)	530.5	(16.5)
missing	0.2	161.2	(48.6)	150.5	(60.0)	311.7	(96.5)
*Education Level*							
Primary	33.6	210.3	(4.4)	296.2	(7.2)	506.5	(8.1)
Secondary	41.6	240.7	(4.5)	277.8	(6.2)	518.6	(7.2)
Tertirary	21.5	253.0	(6.5)	290.7	(8.9)	543.7	(10.1)
Missing	3.3	216.5	(15.8)	347.4	(23.5)	563.9	(28.7)
*Country of Birth*							
Sweden	79.9	231.4	(3.1)	294.5	(4.6)	525.9	(5.2)
Other	20.1	236.3	(6.6)	267.3	(9.1)	503.6	(11.1)
*Living situation*							
Cohabiting	30.2	297.0	(5.9)	170.8	(5.9)	467.9	(8.0)
Living alone	69.8	204.4	(3.0)	340.1	(5.2)	544.6	(5.8)
*Underlying cause of death*							
Cancer-related	23.1	388.5	(6.3)	125.1	(5.1)	513.6	(7.9)
Dementia-related	17.4	103.6	(3.9)	542.7	(12.5)	646.3	(12.9)
Respiratory-related	7.2	285.4	(11.2)	275.1	(14.5)	560.5	(17.5)
Circulatory-related	30.6	174.8	(3.8)	270.6	(7.1)	445.5	(8.1)
Other	21.8	232.7	(7.3)	291.2	(8.6)	523.9	(11.0)
*Place of death*							
Hospital	44.3	329.8	(5.0)	172.2	(4.5)	502.1	(6.7)
Care home/assisted living	39.7	129.1	(3.0)	499.6	(8.1)	628.7	(8.4)
Private residence	13.9	216.7	(7.1)	133.9	(6.6)	350.5	(10.5)
Other	2.0	344.3	(20.6)	91.5	(12.4)	435.8	(24.9)

*(%) the proportion, SEK K= Swedish Kronor in thousands, (conversion rate SEK100=$9.20 or SEK100=€8.64)

The average total care expenditure per individual was SEK521.4 K during the last 12 months of life, healthcare accounted for 44.6% and social care accounted for 55.4% of total care expenditure. Healthcare expenditure decreased whereas social care expenditure increased in older age groups, however, decedents ≥ 90 years incurred the highest average total care expenditure (SEK 595.9 K). On average males incurred higher healthcare expenditure (SEK 264.6 K) compared to females who incurred higher social care expenditure (SEK 345.0 K). Those in the lowest income group incurred lower healthcare and higher social care expenditure compared to higher income groups. Cohabiting decedents had higher average healthcare expenditure (SEK 297.0 K), compared to those living alone who had higher social care expenditure (SEK 340.1K). Care expenditure differed according to underlying cause of death, as cancer-related deaths had on average higher healthcare expenditure (SEK 388.5 K), and dementia-related had on average social care expenditure (SEK542.7 K). In the study, *n* = 252 individuals who incurred zero healthcare expenditure and *n* = 4,721 who incurred zero social care expenditure. There were 177 decedents who incurred zero care expenditure during their last 12 months, these individuals were included in the GBTM analysis.

## Group-Based trajectory modelling (GBTM)

**Fig. 1:**
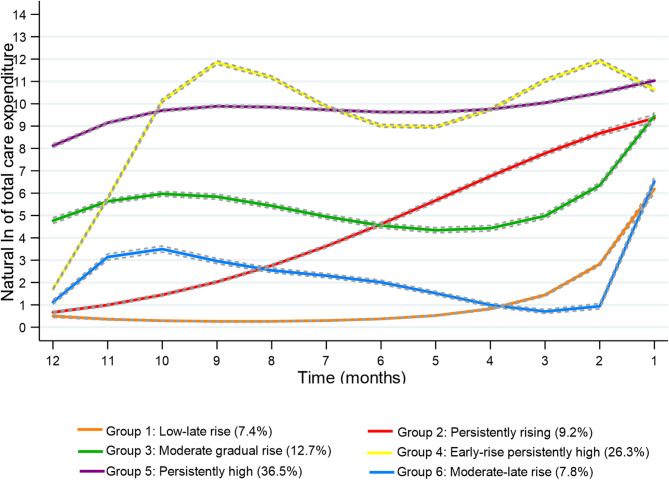
Estimated health and social care expenditure trajectories during the 12-months prior to date of death among (*N* = 12,104) persons 70 years and older that died in Stockholm County in 2019

The GBTM identified six distinct trajectories of total health and social care expenditure within the (*N* = 12,104) study population over the last 12 months, these estimated trajectories and the proportions of the study population assigned to each group are shown in Fig. 1.

Overall, 7.4% of the population followed a trajectory characterized by low expenditure with a late rise towards the last months of life (group 1) illustrated by the orange line in Fig. 1. Individuals who incurred zero care expenditure followed the low-late rise trajectory. Further, 9.2% of decedents followed a trajectory characterized as “persistently rising” (group 2, represented by the red line in Fig. 1), for whom care expenditure was increasing throughout the last 12 months. In contrast, the care expenditure for those following the “moderate gradual rise” expenditure trajectory (group 3), was relatively stable and peaked in the last month of life. The expenditure trajectory indicated by the yellow line in Fig. 1 (group 4) included 26.3% of decedents and this trajectory was characterized as “early-rise persistently high” as their care expenditure increased at the beginning and remained high with some fluctuations until the last month of life. the largest trajectory identified included 36.5% (group 5) of decedents who had “persistently high” care expenditure throughout the last 12-months. Lastly, 7.8% of decedents followed a “moderate, late rise” (group 6), with a slight initial increase in expenditure, followed by a gradual decline, and a sharp rise in the final month.

Table 2 describes the average expenditure on health and social care services per individual stratified by estimated expenditure trajectories identified in GBTM. Decedents who followed the persistently high trajectory (group 5) incurred the highest average expenditure on outpatient care, specialist outpatient care, emergency care, home-healthcare and inpatient care, in addition to the highest average expenditure on home-help services (SEK 175.0 K) in their last 12 months. Compared to the early rise persistently high trajectory (group 4) who incurred the highest average per individual expenditure on care home placement (SEK 532.0 K) and the second highest average expenditure on home-help services (SEK 76.6 K). In contrast, those individuals who followed the low-late rise (group 1) trajectory had the lowest average expenditure for emergency care (SEK 4.5 K), outpatient care (SEK 0.6 K) and specialist outpatient care (SEK 1.1 K) per individual. While the moderate late rise (group 6) had the lowest average total care expenditure (SEK 56.3 K), with the lowest expenditure on social care services. Those that followed the persistently rising trajectory (group 2), incurred the second highest average expenditure inpatient care (SEK 166.9 K).

**Table 2:** The average per individual expenditure on health and social care services for the estimated trajectories

	Mean expenditure in Swedish Kronor in thousands (SEK K)					
	Group 1 (Low-late rise)	Group 2 (Persistently rising)	Group 3 (Moderate-gradual rise)	Group 4 (Early rise, persistently high)	Group 5 (Persistently high)	Group 6 (Moderate, late rise)
	*N* = 893	*N* = 1,129	*N* = 1,525	*N* = 3,209	*N* = 4,401	*N* = 947
	SEK K (SE)	SEK K (SE)	SEK K (SE)	SEK K (SE))	SEK K (SE)	SEK K (SE)
*Total care expenditure*	68.4 (5.3)	270.5 (8.8)	198.1 (8.1)	807.7 (9.1)	681.0 (7.4)	56.3 (3.0)
*Healthcare expenditure*						
Emergency care	4.5 (0.3)	29.4 (1.2)	21.0 (0.6)	29.2 (1.1)	57.3 (1.7)	5.9 (0.3)
Outpatient care	0.6 (0.1)	4.5 (0.2)	5.2 (0.2)	6.5 (0.3)	17.8 (0.4)	1.0 (0.1)
Home-healthcare	1.2 (0.3)	15.0 (1.2)	6.0 (0.5)	21.1 (1.1)	60.6 (1.8)	0.5 (0.1)
Outpatient specialist care	1.1 (0.1)	3.4 (0.2)	5.0 (0.2)	4.1 (0.2)	5.5 (0.2)	2.7 (0.1)
Inpatient care	52.1 (4.8)	166.9 (7.2)	141.2 (8.6)	138.1 (3.6)	211.5 (3.9)	40.9 (2.7)
*Social care expenditure*						
Home-help services	2.2 (0.4)	18.4 (1.7)	10.5 (0.8)	76.6 (3.0)	175.0 (3.9)	1.5 (0.3)
Care home placement	6.6 (1.5)	32.8 (3.5)	9.1 (1.5)	532.0 (10.7)	153.3 (5.7)	3.8 (0.8)

*SEK K=Swedish Kronor in thousands (conversion rate SEK100=$9.20 or SEK100=€8.64), Mean= the average care expenditure, (SE)=standard error

Table 3 describes the socio-economic and demographic composition of the estimated expenditure trajectories. Most individuals in the “low late rise” trajectory (group 1) were female (57.2%), lived alone (76.8%), were in the lowest income group (43.7%) and born outside of Sweden (30.8%) compared to the other trajectories. The “persistently rising” trajectory (group 2) were younger, and cohabiting (36.5%). Most deaths were cancer-related (32.3%) and 48.4% occurred in hospital settings. The “moderate-late rise” trajectory, 34.8% of deaths were circulatory-related. Most of the “early-rise, persistently high” trajectory (group 4) was ≥ 90 years (35.7%), female (59.5%) and (51%) died in care homes or assisted living facilities. Most in the “persistently high” trajectory (group 5) had (23.1%) tertiary education level and the largest proportion of respiratory-related deaths, and 53.3% of deaths occurred in hospitals.

**Table 3:** The socio-economic and demographic composition of the estimated expenditure trajectories

	Group 1 (Low-late rise)	Group 2 (Persistently rising)	Group 3 (Moderate- Gradual rise)	Group 4 (Early-rise, persistently high)	Group 5 (Persistently high)	Group 6 (Moderate late rise)
	*N*=893	*N*=1,129	*N*=1,525	*N*=3,209	*N*=4,401	*N*=947
	(%)	(%)	(%)	(%)	(%)	(%)
*Age group*s						
70-74 yrs	17.9	19.4	14.6	10.7	13.9	11.6
75-79 yrs	18.4	20.5	18.6	13.8	16.4	16.5
80-84 yrs	16.2	21.5	21.4	17.3	19.2	20.7
85-89 yrs	18.8	18.9	23.1	22.5	22.2	21.1
** ≥**90 yrs	28.7	19.8	22.2	35.7	28.3	30.1
*Sex*						
Male	42.8	50.8	52.5	40.5	48.1	42.7
Female	57.2	49.2	47.5	59.5	51.9	57.3
*Income groups*						
(lowest) group 1	49.2	36.7	34.3	39.8	36.2	44.0
group 2	30.2	36.3	35.9	35.0	35.8	32.2
group 3	7.8	12.1	11.9	10.8	12.7	8.7
group 4	5.2	6.1	7.0	6.3	6.8	5.4
(highest) group 5	6.8	8.4	10.6	8.0	8.3	9.4
missing	0.8	0.4	0.3	0.1	0.1	0.3
*Education Level*						
Primary	36.2	34.4	32.5	33.4	32.6	37.2
Secondary	43.7	42.2	42.6	41.7	41.1	39.8
Tertirary	14.9	19.8	22.2	21.9	23.1	19.6
missing	5.3	3.6	2.8	3.0	3.2	3.4
*Country of Birth*						
Sweden	69.2	78.1	80.2	81.0	81.8	79.0
Other	30.8	21.9	19.8	19.0	19.2	21.0
*Living situation*						
Cohabiting	23.3	36.5	35.4	25.6	32.6	24.6
Living alone	76.6	63.5	64.6	74.4	67.4	75.4
*Underlying cause of death*						
Cancer-related	12.5	32.4	21.6	19.9	28.9	7.9
Dementia-related	26.7	13.7	11.6	24.3	10.8	29.3
Respiratory-related	4.8	5.5	7.9	7.3	8.0	5.7
Circulatory-related	28.8	27.5	34.8	28.4	30.4	37.4
Other	27.2	20.8	24.0	20.1	21.8	19.7
*Place of death*						
Hospital	25.8	48.4	46.4	36.0	53.3	21.8
Care home/assisted living	42.9	30.7	33.2	51.0	27.9	57.2
Private home	17.8	13.8	14.8	9.7	14.5	14.4
Other	1.6	2.7	2.2	1.6	2.2	1.8
Missing	12.0	4.3	3.3	1.7	2.1	4.9

*(%) the proportion

Multinominal logistic regression models were performed to assess the factors associated with spending trajectory membership. Model 1 was adjusted for socio-economic and demographic factors presented in Table S3. Decedents following the “low-late rise” trajectory (group 1) were 83% more likely to be born outside of Sweden, 38% more likely to be living alone and to be lowest income group compared to those in the “persistently high” trajectory (group 5). The “early-rise, persistently high” trajectory (group 4) were 22% more likely to be female and older compared to the persistently high trajectory (group 5). Model 2 was adjusted for model 1, underlying cause of death and place of death see Table S4. The “moderate-late rise” trajectory (group 6) were 41% more likely to have had a cardiovascular-related death compared to the “persistently high” trajectory (group 5). While decedents following the trajectories corresponding to group 1, group 4, and group 6 were less likely to have a cancer-related death compared to the “persistently high” trajectory (group 5). In terms of place of death, the “early rise, persistently high” trajectory (group 4) were 1.93 times (CI: 1.48–2.50) more likely to die in care home/assisted living facility. While decedents in group 1, group 2, group 3 and group 6 were significantly less likely to die in a hospital setting compared to the “persistently high” trajectory (group 5).

## Discussion

This study identified six distinct trajectories of health and social care expenditure among those seventy years and older who died in Stockholm County in 2019. The largest expenditure trajectory was characterized as having a “persistently high” pattern of care expenditure, incurring the highest average expenditure on all healthcare (inpatient and outpatient) as well as home-help services during their last year of life. The second largest trajectory was characterized as “early rise, persistently high”, and individuals following this trajectory incurred the highest average expenditure for care home placements. In the multinomial logistic regression models, those in the “low-late rise” trajectory were more likely to be born outside of Sweden, to live alone, and be in lower income groups compared to those in the “persistently high” spending trajectory. Additionally, those in the “early-rise, persistently high” trajectory was more likely to be female, higher age, with a dementia-related cause of death, and to die in a care home or assisted living facility. Further, those in the “moderate-late rise” expenditure were more likely to die from cardiovascular diseases compared to the “persistently high” trajectory.

The health and social care expenditure trajectories identified in this study are comparable to previous studies exploring patterns of care utilization and expenditure among older persons at the end-of-life [6, 9, 21]. A Japanese study identified six trajectories of health and social care expenditure among persons sixty-five years and older in the five years before death. However, despite the longer follow-up period the authors were unable to pinpoint the onset of rising care expenditure because of the complex care needs and multiple chronic conditions [9]. A similar Danish study identified four expenditure trajectories where the expenditure measure included hospital treatments, prescription drugs, primary care visits, and community-based services. The largest trajectories identified were characterized as high and moderately high healthcare expenditure, and the number of chronic conditions being the main predictor. Moreover, consistent with our findings, older age was associated with increased social care and decreased inpatient care expenditure [6]. Likewise, a Swedish study by Ebeling et al., identified six trajectories of care utilization, measured as days spent in inpatient care and specialist outpatient visits, as well as whether social care was used during the last year of life [21]. The largest utilisation trajectories identified were comparable to the “persistently high” and “early rise, persistently high” expenditure trajectories, and were distinguished by having high expenditure on home-help services and care home placements. However, Ebeling et al.‘s study lacked measures of outpatient care services, such as primary care and home-healthcare services, which are crucial to understand the care resources needed for the management chronic conditions and provision of home-based care [21]. Care expenditure is more informative as a comprehensive measure of the care resources used in the last year of life, capturing both the quantity and intensity of care services involved, which provides insight into where more resources will be needed as the population ages.

The care services utilized by the “persistently high” expenditure trajectory were largely healthcare services (inpatient, emergency, outpatient, and home-healthcare) financed by the region and also had the highest average expenditure on home-help services financed by municipalities. The Swedish healthcare system is subdivided into specialized services focused on treating and managing specific conditions rather than addressing the entirety of the care needs experienced by an individual. Additionally, there is limited coordination with municipal social care services which may compromise patient care experience, lead to inefficiencies, and finally lead to higher care costs for both regions and municipalities. The care needs of the “persistently high” trajectory were complex, with a large quantity and high intensity of services required from multiple regional and municipal care providers. Moreover, individuals following this expenditure trajectory seem to be a good example of “ageing in place”, given the high amount of home-based care required to support them in their last year of life. Additionally, high expenditure on emergency care adds to increasing inpatient care expenditure, as emergency department visits among this age group often lead to unplanned hospitalizations, with long inpatient stays and additional treatments [34]. Although it is possible that the home-based care received does not sufficiently meet the care needs of individuals in the “persistently high” trajectory, a systematic review reported that receiving appropriate home-based palliative care was associated with lower emergency care use among dying patients [35]. Most decedents lived alone which may lead to difficulties in navigating complex care systems in conjunction with experiencing poor care coordination resulting in higher care expenditure. However, older people might supplement formal care services with informal care from family and friends which will potentially become an increasingly important care resource in the future.

This study observed socio-economic and demographic differences in end-of-life care expenditure, and previous research found that higher socio-economic groups incur higher care expenditure to varying extents in different countries [16]. Previous Swedish studies have observed that lower socio-economic groups incur lower healthcare expenditure in the last year of life [15, 36]. Those following the “low-late rise” trajectory were comparatively more disadvantaged, with most being in the lowest income quintile and with the largest proportion of migrants. Lower total care expenditure in the last year of life could be attributed to experiencing fewer health problems and care needs, or due to a sudden death [11]. However, incurring low care expenditure during a time when needs tend be higher could be attributed to encountering barriers to accessing care and consequently, producing unmet care needs [37]. Further, this study observed sex differences in the care expenditure, where males incurred higher average healthcare expenditure, and conversely females had higher social care expenditure, consistent with previous research [6, 9]. This variation in care expenditure could be because females on average live longer than males but tend to experience more chronic and disabling diseases while males experience more severe diseases resulting in higher mortality [38]. The composition of expenditure trajectories reflected sex differences, as more males followed the “persistently rising” and “moderate, gradual rise” groups, both characterized as having high expenditure on healthcare services. Moreover, among these there were many cancer and circulatory-related deaths, both of which entail frequent treatment and follow-up within healthcare. Moreover, there were comparatively more individuals cohabiting within these trajectories, which might indicate having access to sources of informal care, which may partially alleviate social care needs. On the other hand, the care expenditure of female decedents following the “early rise, persistently high” trajectory, was predominantly on social care services, and particularly on care home placements. Decedents following this trajectory were on average older and most deaths were dementia related. Generally, females at higher ages tend to reside in care homes more often than males, and dementia is an important predictor of care home admission [39] and disproportionately affects females [40]. Highlighting the socio-economic and demographic differences in care expenditure informs more equitable and efficient future care planning by identifying groups with varying needs and resource use.

Most older people would prefer to receive care and die in their own homes [41]. Generally, this preference to remain in your own home is stable, as conditions progress and illnesses become increasingly severe. The stability of care preferences has been illustrated by a Swedish study where the priorities of community-based older people remained consistent over time [42]. Research conducted previously revealed that trends in the place of death changed in Sweden between 2013 and 2019, where overall the proportion of deaths occurring in private residences increased and hospital deaths decreased, though this varied greatly between regions [43]. Moreover, the authors found that individuals living in municipal care homes had a decreased likelihood of dying in hospital compared to those in their own home [43]. In this study population most deaths occurred in hospital settings, although this varied between expenditure trajectories, as the “low-late rise” trajectory had the largest proportion of deaths occurring in private homes, while most deaths among those following the “early rise, persistently high” occurred in care homes/assisted living facilities, and accordingly those following this trajectory incurred the highest average expenditure on municipal care homes. Variation in place of death observed between the expenditure trajectories highlights the complexity of end-of-life care needs. Insights into where people die, in conjunction with their patterns care resource use, is essential for planning palliative care services.

The composition of care services consumed by the “early rise, persistently high” group was not restricted to social care, as they also incurred high expenditure on healthcare services. A Swedish study examined the external medical care utilized by care home residents in the last year of life between 2015 and 2021, and observed that male and younger (65–79 years) residents incurred higher healthcare expenditure, compared to female and older residents [44]. Further, an advanced cancer diagnosis or high-risk frailty score was associated with an increased risk of high medical costs, alternatively having a dementia diagnosis was associated with a lower risk of medical costs. Further, these findings remained stable throughout the last year of life, even among those that did not reside in a municipal care home for the entire period [44]. Typically, Swedish care homes are staffed with assistant nurses and support staff who have less formal training and competence for providing end-of-life care. Hence, care home residents have an increased likelihood of hospitalization during the last year of life [44]. Considering that hospital-based inpatient care and care home placements are the most expensive types of care, this might allude to an area of insufficiencies in either governance, organization, or staff competencies.

### Strengths and limitations

While the identified trajectories have been examined to support their plausibility, they should be thought of as a way to decompose the variability of the population care expenditure trajectories rather than as “real” sub-populations [30]. Additionally, the expenditure of individuals within the same trajectory can vary widely around the mean and have different proportions of the various care services. The generalisability of these findings should be carefully considered, given that there are different approaches to organising the delivery of and regulating the access to health and social care services, in other countries [4]. There are challenges to generalizing these findings within Sweden, as the regions and municipalities are autonomous, and decide how care resources should be distributed depending on the demographic composition and needs of the local population [19, 43]. An advantage of having this study set in Region Stockholm is the ability to include measures of expenditure on primary care and home-healthcare services, as the municipalities are responsible for providing home-healthcare services in the other regions [19]. Additionally, information on primary care utilisation is not available in the national patient registers, yet our findings are comparable to studies based on national data [21].

Income quintiles were calculated based on the equivalised disposable income distribution of the entire adult population, in line with national standards and to reflect the shared economic context in which individuals aged 70 and above live [24]. This approach can be considered a strength as it captures structural inequalities and situates older adults within the broader societal distribution of economic resources. At the same time, this measurement approach has potential limitations, as income and age may still be correlated with the older population, which could confound associations with care expenditure. Though this was addressed in the analysis by adjusting models by age.

It was not possible to assess the individual experience of care in terms of whether the care used, and treatments administered, were aligned with the wishes of the individual. Further, this study did not include a measure of the number of chronic conditions, which may be a limitation considering that the number of chronic conditions has been found to be significantly associated with higher healthcare costs [6, 9]. The underlying cause of death was included as it directly relates to the terminal events and is associated with the type of care used at the end-of-life [11, 21, 45]. However, in older adults that experience multiple chronic conditions it can be difficult to discern the exact cause of death. Additionally, underlying cause of death may be missing when there is limited information, and the death will then be recorded as sudden/ill-defined [25]. In this study, there was a small number of persons recorded as having ill-defined or accidental deaths, though these types of deaths did not cluster in any specific expenditure trajectory.

This study included a wide range of inpatient and outpatient care services and social care services, but the care expenditure measure was not complete, as a measure of expenditure on prescription drugs could not be included, and previous studies have observed prescription drugs are a driver of high end-of-life expenditure [6, 7]. Despite having a comprehensive system of publicly funded health and social care services in Sweden, informal care typically provided by spouses and/or adult children remains an important complement and may substitute formal care services, particularly for older adults living in their own homes. However, it was beyond the scope of this study to measure access to informal care, which is essential to reflect upon when interpreting the findings, as access to informal care could influence the shape and pattern of the identified expenditure trajectories. A consequence of not having a measure of the contribution from informal caregivers may be an underestimation of the total care burden which could obscure socio-economic or demographic differences in end-of-life support. In cases where informal care substitutes for formal services, it could result in lower expenditure on formal care services during the last year of life. For example, individuals with strong informal support networks may use fewer formal care services (i.e. those that had zero care expenditure), potentially appearing in the lower-expenditure trajectory groups identified in this study. Conversely, those without access to informal care may rely more heavily on formal services, leading to higher health and social care expenditures.

## Conclusion

This study observed distinct patterns of care expenditure among older persons at the end-of-life and multiple factors contributed to these patterns. Most followed a trajectory where both health and social expenditure remained high and were relying on care in their own homes. As individuals approach death, health deteriorates and illnesses accumulate, the number of actors from health and social care services involved in care tends to increase and become more expensive. These findings provide insight into the health and social care resources being used during a care-intensive period of life, which can inform policies on care planning and resource allocation to meet the care needs of an ageing population.

## Supplementary Information

Supplementary Material 1.

## Data Availability

The data used to perform this study cannot be made available upon request. In accordance with the General Data Protection Regulation, The Swedish law SFS 2018:218, The Swedish Data Protection Act, the Swedish Ethical Review Act, and the Public Access to Information and Secrecy Act, these types of sensitive data can only be made available after legal review, for researchers who meet the criteria for access to this type of sensitive and confidential data. Readers may contact the first author regarding any further details.

## Abbreviations

SEK: Swedish Kronor
SEK K: Swedish Kronor in thousands
RTB: Total Population Register
LISA: The Longitudinal Integrated Database for Social Insurance and Labour Market Studies
VAL: Region Stockholm Healthcare Utilization Administrative Database
DRG: Diagnosis Related Groups
GBTM: Group Based Trajectory Modelling
RRR: Relative Risk Ratio
CI: Confidence Interval
