# Supplementary material for: Trajectories of health and social care expenditure in the last year of life among people 70 years and older in Region Stockholm: a population-based cohort study
Source: BMC Geriatr. 2025 Nov 4;25:838. doi: 10.1186/s12877-025-06498-0 (PMC12584503; doi:10.1186/s12877-025-06498-0)
Supplement: Supplementary file 1 — Supplementary Material 1. [file 12877_2025_6498_MOESM1_ESM.docx]

# Supplementary material

**Table S1** Classification accuracy and model fit statistics for six-group trajectory model

|  | Number of observations | Average posterior propability (APP) | Odds of correct classification (OCC) | Odds of correct classification based on the posterior probability | Observed proportion in assigned to each group | Total probability |
| --- | --- | --- | --- | --- | --- | --- |
| Group 1 | 893 | 0.966436 | 361.481 | 359.1922 | 0.073777 | 0.074213 |
| Group 2 | 1129 | 0.953229 | 198.1195 | 200.0267 | 0.093275 | 0.092468 |
| Group 3 | 1525 | 0.933871 | 97.96534 | 96.9081 | 0.125991 | 0.127191 |
| Group 4 | 3209 | 0.974884 | 107.5934 | 108.7344 | 0.265119 | 0.263069 |
| Group 5 | 4401 | 0.984847 | 113.754 | 113.0161 | 0.363599 | 0.365106 |
| Group 6 | 947 | 0.946328 | 207.7252 | 208.5489 | 0.078239 | 0.077954 |

The model fitted has a high APP for each group, indicating strong confidence in group assignments. The observed and the expected proportion align closely, which indicates that the predicted group sizes fit well with the observed data. Overall, the six-group model fits the data well with clear and distinct classifications for each trajectory group.

**Table S2:** Comparison of average care expenditure (expressed as Swedish kronor in thousands in identified spending trajectories within 95% confidence intervals**.**

|  | **Health care expenditure** | | | **Social care expenditure** | | | **Total care expenditure** | | |
| --- | --- | --- | --- | --- | --- | --- | --- | --- | --- |
|  | **Mean (SEK K)** | **SE** | **CI 95%** | **Mean (SEK K)** | **SE** | **CI 95%** | **Mean (SEK K)** | **SE** | **CI 95%** |
| Group 1 (Low-late rise) | 59.6 | 5.0 | 49.8–69.4 | SEK 8.9 | 1.6 | 5.8–12.0 | 68.5 | 5.3 | 58.0–79.0 |
| Group 2 (Persistently rising) | 219.2 | 8.0 | 203.6–235.0 | SEK 51.2 | 3.8 | 43.8–58.7 | 270.5 | 8.8 | 253.1–287.8 |
| Group 3 (Moderate, gradual rise) | 178.5 | 8.8 | 161.4–195.7 | SEK 19.6 | 1.7 | 16.3–22.9 | 198.1 | 8.9 | 180.6–215.6 |
| Group 4 (Early-rise, persistently high) | 199.1 | 4.8 | 190.2–208.1 | SEK 608.5 | 10.0 | 588.9–628.2 | 807.7 | 9.1 | 789.8–825.5 |
| Group 5 (Persistently high) | 352.7 | 5.2 | 342.5–362.8 | SEK 328.4 | 6.2 | 316.2–340.6 | 681.0 | 7.4 | 666.5–695.6 |
| Group 6 (Moderate, late-rise) | 51.0 | 2.9 | 45.4–56.7 | SEK 5.3 | 0.9 | 3.6–7.0 | 56.3 | 3.0 | 50.4–62.3 |

*SEK K=Swedish kronor in thousands, CI=95% confidence interval, SE=standard error

**Table S3:** Multinominal logistic regression examining the effect of demographic and socio-economic factors associated with membership to expenditure trajectory groups.

|  | **Group 1 (Low-late rise)** | | **Group 2 (Persistently rising)** | | **Group 3 (Moderate, gradual rise)** | | **Group 4 (Early-rise, persistently high)** | | **Group 6 (Moderate, late-rise)** | |
| --- | --- | --- | --- | --- | --- | --- | --- | --- | --- | --- |
|  | **RRR** | **CI 95%** | **RRR** | **CI 95%** | **RRR** | **CI 95%** | **RRR** | **CI 95%** | **RRR** | **CI 95%** |
| ***Sex*** |  |  |  |  |  |  |  |  |  |  |
| Male | ref |  | ref |  | ref |  | ref |  | ref |  |
| female | 1.09 | 0.93-1.27 | 0.97 | 0.84-1.12 | 0.89 | 0.78-1.00 | 1.22 | 1.11-1.35 | 1.09 | 0.94-1.27 |
| ***Age groups*** |  |  |  |  |  |  |  |  |  |  |
| 70–74 yrs | ref |  | ref |  | ref |  | ref |  | ref |  |
| 75–79 yrs | 0.85 | 0.66-1.08 | 0.89 | 0.72-1.10 | 1.09 | 0.89-1.35 | 1.08 | 0.90-1.29 | 1.20 | 0.92-1.56 |
| 80–84 yrs | 0.63 | 0.49-0.80 | 0.79 | 0.64–0.98 | 1.09 | 0.89-1.33 | 1.14 | 0.96-1.36 | 1.27 | 0.98–1.64 |
| 85–89 yrs | 0.61 | 0.48-0.77 | 0.60 | 0.48-0.75 | 1.02 | 0.84-1.25 | 1.25 | 1.06-1.48 | 1.09 | 0.85-1.41 |
| 90+ yrs | 0.70 | 0.56-0.88 | 0.51 | 0.41-0.63 | 0.79 | 0.65–0.97 | 1.49 | 1.27-1.74 | 1.14 | 0.89-1.46 |
| ***Country of birth*** |  |  |  |  |  |  |  |  |  |  |
| Sweden | ref |  | ref |  | ref |  | ref |  | ref |  |
| Other | 1.83 | 1.55-2.16 | 1.20 | 1.02-1.41 | 1.11 | 0.95-1.29 | 1.07 | 0.95-1.20 | 1.13 | 0.95-1.36 |
| ***Living situation*** |  |  |  |  |  |  |  |  |  |  |
| Cohabiting | ref |  | ref |  | ref |  | ref |  | ref |  |
| alone | 1.38 | 1.14-1.66 | 0.90 | 0.77-1.05 | 0.97 | 0.85-1.12 | 1.22 | 1.09-1.37 | 1.33 | 1.10-1.59 |
| ***Income groups*** |  |  |  |  |  |  |  |  |  |  |
| (Highest) group 5 | ref |  | ref |  | ref |  | ref |  | ref |  |
| (Lowest) group 1 | 1.33 | 0.98–1.80 | 1.11 | 0.85-1.44 | 0.79 | 0.63–0.98 | 0.96 | 0.80-1.15 | 0.92 | 0.70-1.20 |
| group 2 | 1.01 | 0.75-1.37 | 1.09 | 0.84-1.40 | 0.80 | 0.65–0.99 | 0.96 | 0.80-1.15 | 0.75 | 0.58-0.98 |
| group 3 | 0.78 | 0.54-1.13 | 0.95 | 0.71-1.27 | 0.73 | 0.57-0.94 | 0.91 | 0.74-1.12 | 0.61 | 0.44-0.85 |
| group 4 | 0.94 | 0.62-1.42 | 0.86 | 0.61-1.22 | 0.79 | 0.59-1.06 | 1.01 | 0.79-1.29 | 0.72 | 0.49-1.05 |

Model 1: sex, age group, country of birth, living situation and income group. Notes: N=12,079 observations. The reference group for the outcome variable was the “Persistently High” expenditure trajectory group 5. *RRR = relative risk ratios, CI=confidence intervals, ref=reference category.

**Table S4:** Multinominal logistic regression examining the effect of demographic, socio-economic factors, underlying cause of death and place of death associated with membership to expenditure trajectory groups.

|  | **Group 1 (Low-late rise)** | | **Group 2 (Persistently rising)** | | **Group 3 (Moderate, gradual rise)** | | **Group 4 (Early-rise, persistently high)** | | **Group 6 (Moderate, late-rise)** | |
| --- | --- | --- | --- | --- | --- | --- | --- | --- | --- | --- |
|  | **RRR** | **CI 95%** | **RRR** | **CI 95%** | **RRR** | **CI 95%** | **RRR** | **CI 95%** | **RRR** | **CI 95%** |
| ***Sex*** |  |  |  |  |  |  |  |  |  |  |
| Male | ref |  | ref |  | ref |  | ref |  | ref |  |
| female | 1.07 | 0.91-1.26 | 0.94 | 0.82-1.08 | 0.89 | 0.78-1.01 | 1.14 | 1.03-1.26 | 1.03 | 0.88-1.21 |
| ***Age groups*** |  |  |  |  |  |  |  |  |  |  |
| 70–74 yrs | ref |  | ref |  | ref |  | ref |  | ref |  |
| 75–79 yrs | 0.81 | 0.63-1.05 | 0.89 | 0.71-1.10 | 1.05 | 0.86-1.30 | 0.99 | 0.83-1.19 | 1.06 | 0.80-1.39 |
| 80–84 yrs | 0.52 | 0.40-0.68 | 0.76 | 0.61–0.94 | 0.99 | 0.80-1.21 | 0.92 | 0.78-1.10 | 0.89 | 0.68-1.16 |
| 85–89 yrs | 0.47 | 0.36-0.61 | 0.56 | 0.45-0.70 | 0.88 | 0.72-1.08 | 0.92 | 0.78-1.10 | 0.66 | 0.50-0.87 |
| 90+ yrs | 0.48 | 0.37-0.61 | 0.46 | 0.37-0.58 | 0.62 | 0.50-0.77 | 0.97 | 0.82-1.15 | 0.56 | 0.43-0.73 |
| ***Country of birth*** |  |  |  |  |  |  |  |  |  |  |
| Sweden | ref |  | ref |  | ref |  | ref |  | ref |  |
| Other | 1.69 | 1.42-2.01 | 1.19 | 1.01-1.41 | 1.10 | 0.95-1.28 | 1.10 | 0.97-1.24 | 1.12 | 0.93-1.35 |
| ***Living situation*** |  |  |  |  |  |  |  |  |  |  |
| Cohabiting | ref |  | ref |  | ref |  | ref |  | ref |  |
| Living alone | 1.24 | 1.02-1.51 | 0.89 | 0.76-1.04 | 0.93 | 0.80-1.07 | 1.14 | 1.01-1.28 | 1.13 | 0.94-1.36 |
| ***Income groups*** |  |  |  |  |  |  |  |  |  |  |
| (Highest) group 5 | ref |  | ref |  | ref |  | ref |  | ref |  |
| (Lowest) group 1 | 1.35 | 0.99–1.85 | 1.15 | 0.88-1.50 | 0.78 | 0.62–0.97 | 1.02 | 0.84-1.23 | 0.96 | 0.73-1.27 |
| group 2 | 1.05 | 0.77-1.43 | 1.13 | 0.88-1.46 | 0.80 | 0.64–0.99 | 1.02 | 0.85-1.22 | 0.80 | 0.61-1.05 |
| group 3 | 0.82 | 0.56-1.19 | 0.97 | 0.72-1.30 | 0.74 | 0.58-0.96 | 0.96 | 0.78-1.19 | 0.66 | 0.47-0.94 |
| group 4 | 0.99 | 0.65-1.51 | 0.88 | 0.62-1.24 | 0.83 | 0.62-1.10 | 1.08 | 0.84-1.38 | 0.79 | 0.53-1.16 |
| ***Underlying cause of death*** |  |  |  |  |  |  |  |  |  |  |
| Cancer-related | 0.41 | 0.32-0.52 | 1.10 | 0.91-1.34 | 0.65 | 0.54-0.77 | 0.87 | 0.76-1.01 | 0.36 | 0.27-0.48 |
| Dementia-related | 2.20 | 1.73–2.80 | 1.31 | 1.02-1.69 | 0.85 | 0.68-1.07 | 1.65 | 1.40-1.94 | 2.11 | 1.67–2.68 |
| Respiratory-related | 0.63 | 0.44-0.90 | 0.74 | 0.54-1.01 | 0.92 | 0.72-1.17 | 1.07 | 0.88-1.31 | 0.96 | 0.69-1.34 |
| Cardiovascular-related | 0.90 | 0.74-1.11 | 1.01 | 0.83-1.23 | 1.06 | 0.90-1.24 | 0.99 | 0.87-1.13 | 1.41 | 1.15-1.73 |
| Place of death |  |  |  |  |  |  |  |  |  |  |
| hospital | 0.19 | 0.14-0.25 | 0.61 | 0.46-0.81 | 0.72 | 0.54-0.95 | 0.88 | 0.69-1.14 | 0.27 | 0.19-0.38 |
| Care home/living facility | 0.41 | 0.31-0.56 | 0.84 | 0.62-1.15 | 1.07 | 0.80-1.44 | 1.93 | 1.48-2.50 | 1.00 | 0.71-1.39 |
| Private residence | 0.43 | 0.32-0.58 | 0.60 | 0.44-0.83 | 0.78 | 0.58-1.06 | 0.88 | 0.66-1.16 | 0.56 | 0.40-0.80 |

Model 2=Model 1 + underlying cause of death+ place of death. Notes: N=12,079 observations. The reference group for the outcome variable was the “Persistently High” expenditure trajectory group 5. *RRR = relative risk ratios, CI=confidence intervals, ref=reference category.
